# Hygiene of the Mouth

**Published:** 1897-02

**Authors:** 


### Editorial, Etc.

### HYGIENE OF THE MOUTH.

The following editorial in the Philadelphia Polyclinic is presumably written by Prof. Henry Leffmann. Although a chemist and not a practicing dentist, Dr. Leffmann's interest in dental literature and dental meetings makes what he writes and says always welcome. It was our privilege two years ago to hear him discuss the teaching in dental colleges and his views made a favorabie and lasting impression. R. G.

"Side by side with medical science has grown up the profession of dentistry, which is really a specialty of medicine, but has always been independent. The medical degree is not necessary to entitle dentists to practice and medical schools have given but little attention to the study of that portion of anatomy which is the dentist's province. It is, however, now clearly evident that the teeth are as important objects of medical study as the eye or ear. Dental literature, especially the clinical literature, offers abundant evidence of the injustice done to patients in consequence of physicians overlooking the local and general effects of diseased or defective teeth. A considerable proportion of facial neuralgias is merely irregular reflexes of irritation from decayed or inflamed teeth. In neuralgias affecting one side of the head, and especially around the region of the ear, a careful search for diseased teeth should be made. Patients affected with such neuralgias often suffer for a long while in consequence of the cause of the trouble being not diagnosed. They are put on general treatment, taking all the anti-neuralgia remedies, both old standbys and "new fads," and their general health is injured, not only by the use of the drugs, but by the continued suffering. Sometimes relief comes by accident, in consequence of a visit to a dentist who discovers the diseased tooth. Even when these localized neuralgias do not develop, the presence of decayed teeth may lead to stomach troubles in consequence of defective mastication. Pain occasioned by eating leads either to bolting solid food or to the use of such materials as need no chewing, and either of these habits will disturb the functions of the digestive organs.

Another source of danger from decayed teeth is the possible introduction of parasites into the tissues with which the teeth are connected. Parasitic organisms are numerous in articles of food, both as usual and occasional associates, and as it is very difficult to prevent small particles of food from lodging in the cavities of carious teeth and there undergoing decomposition, it is not impossible that by such means, especially if the cavity is the root-channel of a dead tooth, a parasite might enter the soft tissues. In a case that was operated on at the Polyclinic about a year ago there was a suspicion that such a method of infection had occurred. A young man had suffered frequently from swelling of the lymphatic glands on one side of the neck, but the affection had never gone on to the suppurative stage. One attack, however, seemed so threatening that he had the involved gland removed. The operation was a little more extensive than had been anticipated, and a microscopic examination of the mass showed a stellate organism, not a bacterium, the exact nature of which was not made out. It is possible that this was an organism that finds an intermediate host in some animal or plant used as food and that it had entered the system through a tooth-cavity.

It appears from these considerations that full attention should be given to the care of the mouth and the preservation of the teeth. It may be said that no one doubts this or disputes it, but the point we desire to make here is that this is a matter upon which the doctor should be alert to give advice. The same spirit which has led to enlisting the active co-operation of physicians concerning measures for preventing the occurrence of ophthalmia, or the spread of the contagious diseases of childhood, should lead to insisting upon the care of the teeth. It has been well said by a dentist, that while the practice of medicine is confined to the sick the practice of dentistry includes every one from early infancy to the end of life.

The care of the mouth involves more or less operative procedure, but there is general hygiene which each individual should carry out. It is now well established that the cause of tooth-decay is the action of microbes promoted by decomposing food. The use of the tooth-brush after each meal is necessary, but it is not alone sufficient; mild antisiseptic washes are also needed.

It is apparent that medical graduates should be more thoroughly informed as to the nature and relations of the teeth. The instruction in operative procedures and in the manufacture of dental substitutes may properly be left to the dental schools, but every medical college should include in its curriculum a course by practising dentists on the histology and pathology of the teeth.

